# Task-Evoked Negative BOLD Response in the Default Mode Network Does Not Alter Its Functional Connectivity

**DOI:** 10.3389/fncom.2018.00067

**Published:** 2018-08-20

**Authors:** Qolamreza R. Razlighi

**Affiliations:** ^1^Department of Neurology, Collage of Physician and Surgeons, Columbia University, New York, NY, United States; ^2^Taub Institute for Research on Alzheimer's Disease and The Aging, Columbia University, New York, NY, United States; ^3^Biomedical Engineering Department, Columbia University, New York, NY, United States

**Keywords:** negative BOLD response, functional connectivity, Default mode network (DMN), visual network, task-evoked activity, fMRI BOLD, functional neuroimaging (fMRI), brain large-scale networks

## Abstract

While functional connectivity networks are often extracted from resting-state fMRI scans, they have been shown to be active during task performance as well. However, the effect of an in-scanner task on functional connectivity networks is not completely understood. While there is evidence that task-evoked positive BOLD response can alter functional connectivity networks, particularly in the primary sensorimotor cortices, the effect of task-evoked negative BOLD response on the functional connectivity of the Default mode network (DMN) is somewhat ambiguous. In this study, we aim to investigate whether task performance, which is associated with negative BOLD response in the DMN regions, alters the time-course of functional connectivity in the same regions obtained by independent component analysis (ICA). ICA has been used to effectively extract functional connectivity networks during task performance and resting-state. We first demonstrate that performing a simple visual-motor task alters the temporal time-course of the network extracted from the primary visual cortex. Then we show that despite detecting a robust task-evoked negative BOLD response in the DMN regions, a simple visual-motor task does not alter the functional connectivity of the DMN regions. Our findings suggest that different mechanisms may underlie the relationship between task-related activation/deactivation networks and the overlapping functional connectivity networks in the human large-scale brain networks.

## Introduction

Recent advances in the acquisition and analysis of functional magnetic resonance image (fMRI) have made it possible to discover numerous large-scale brain networks based on their spontaneous, but synchronized, low frequency fluctuations at resting-state, as well as during task performance (Biswal et al., 1995; Greicius et al., 2003; Greicius and Menon, 2004; Power et al., 2011; Smith et al., 2013; Cole et al., 2014). Functional connectivity (FC) analyses extract these networks solely based on their interregional temporal coherence, and independently of whether the subject is engaged in a task or is resting (Friston, 1994; Biswal et al., 2010; Di et al., 2013). Alternatively, positive and negative blood oxygenation level dependent (BOLD) signal, which gives rise to activation/deactivation networks, can only be extracted from task-based fMRI (tb-fMRI) scans, and is often based on the time-course of the task being performed. Both task-evoked BOLD response and FC networks during task and rest have been used to investigate the functional architecture of the human brain (Cole et al., 2014; Krienen et al., 2014; Stern et al., 2014). However, some studies have shown that functional architecture identified during resting-state fMRI (rs-fMRI) will give rise to all the possible task-related activation networks; suggesting that tb-fMRI may not be necessary to investigate the functional organization of the human brain (Fair et al., 2007; Fox and Raichle, 2007; Vincent et al., 2007). Cognitive networks such as the default mode, dorsal-attention, fronto-parietal, salience, and executive networks have frequently been detected by using rs-fMRI scans to extract them as resting-state FC (rs-FC) networks (Damoiseaux et al., 2006) and less frequently by using tb-fMRI scans to extract them as task-based FC (tb-FC) networks (Cole et al., 2010; Barch et al., 2013). Furthermore, the convenience of acquiring rs-fMRI scans has resulted in their greater popularity in the field of human neuroimaging, such that the rs-FC networks have become the standard terminology to refer to FC networks. Despite the significant number of studies investigating these rs-FC networks, the effect or influence of task performance on the FC networks is not completely understood. While some studies report a high spatial correspondence between the rs-FC and tb-FC networks (Fox et al., 2006; Fair et al., 2007; Toro et al., 2008; Smith et al., 2009), others claim there are substantive differences between them (Buckner et al., 2013; Hermundstad et al., 2013; Mennes et al., 2013). Gaining a more complete understanding of the accordance and discordance between rs-FC networks and tb-FC networks, therefore, is crucial toward estimating the true underlying functional architecture of the human brain.

In this study our main focus is on the Default mode network (DMN), which is perhaps the most studied large-scale brain network (Bluhm et al., 2008; Buckner et al., 2008). Discovery of the “default mode” in the human brain, dating back two decades, was based on the observation of consistent decreases in cerebral blood flow in a set of brain regions during engagement in a wide range of goal-oriented tasks (Shulman et al., 1997; Raichle et al., 2001). This mechanism of decreases in cerebral blood flow in the DMN is also thought to underlie the task-evoked negative BOLD response that is often detected in the same regions when subjects are engaged in a variety of tasks (Lustig et al., 2003; Pihlajamäki and Sperling, 2009; Sperling et al., 2009). The DMN plays a pivotal role in understanding brain resting state activities, as these regions are shown to have a higher metabolic rate of oxygen and glucose at rest (Vaishnavi et al., 2010; Lu et al., 2011; Spetsieris et al., 2015; Oh et al., 2016). It is also hypothesized that engagement in any task will suppress resting state activity in these regions, and the metabolic resources will instead be redirected to the regions involved in task performance (Raichle et al., 2001). However, the relationship between the task-evoked negative BOLD response in DMN regions and their FC networks during task or at rest is not completely understood (Greicius and Menon, 2004).

Multivariate techniques such as independent component analysis (ICA) have been shown to consistently detect numerous FC networks throughout the entire brain at rest as well as during task performance (McKeown et al., 1998b; Greicius et al., 2003). While studies frequently demonstrate a high spatial correspondence between the rs-FC networks and the tb-FC networks (Fox et al., 2005; Cole et al., 2014; Krienen et al., 2014), there is currently no consensus about the temporal characteristics of these overlapping FC networks. For example, in a simple visual stimulation task, the tb-FC networks in the primary visual cortex show clear modulation by task performance (McKeown et al., 1998a; McKeown, 2000). This modulation is so strong that some studies claim that extracting task-evoked BOLD response can be performed more effectively with FC analysis (i.e., ICA) than with conventional general linear modeling (GLM) analysis, which requires information about the time-course of the tasks (Xu et al., 2013). However, such strong task-related modulation in the time-course of the DMN FC has not usually been reported, even though there is almost a perfect overlap in the spatial pattern of the DMN tb-FC, rs-FC and task-evoked negative BOLD response (Greicius and Menon, 2004). This is rather striking because the existence of overlapping task-evoked negative BOLD response in DMN regions has been frequently reported in the literature (Lustig et al., 2003; Pihlajamäki and Sperling, 2009). The main goal of this study is to investigate whether or not task-evoked negative BOLD response alters the temporal characteristics of the tb-FC network when both are extracted from the DMN regions. Answering this question not only provides crucial information about the functional architecture of the DMN, but also sheds some light on the ongoing debate about the relationship between rs-FC and tb-FC networks.

To investigate the task-related alteration in the temporal characteristics of the tb-FC network extracted from DMN regions, we used a simple visual-motor task, a low-level sensorimotor task consisting of a flashing checkerboard and button press response. Using conventional GLM analysis, we first obtained the task-evoked BOLD response in the primary visual cortex as well as in the DMN regions. Then, we removed the task-related variability from the tb-fMRI data; henceforth called *residualized* tb-fMRI data. Next, FC analyses were performed using ICA on both original tb-fMRI as well as residualized tb-fMRI data. We demonstrate here that removing task-related activity from the tb-fMRI data significantly alters the temporal/spatial characteristics of the tb-FC networks extracted from primary visual cortex, whereas it has no significant effect on the temporal/spatial characteristics of the tb-FC networks extracted from DMN regions, thus providing evidence that task-evoked negative BOLD response does not alter overlapping FC in DMN regions.

## Materials and methods

### Participants

Our dataset included 30 healthy, young, right-handed participants (age = 25 ± 3.5 years, m/f = 10/20) recruited from the Columbia University Medical Center. All participants signed informed consent documents before scanning, and were compensated for their time spent participating in the study. The recruitment procedure, and the experimental design used in this study were approved by Columbia University institutional review board.

### fMRI experimental design

Subjects were presented with visual/audio stimuli (flashing checkerboard/alternating tone) with random onsets (at least 55 events spaced at mean = 6.2 s, range = 4 ~ 18 s) and durations (mean = 1.2 s, range = 0.5 ~ 3.5 s). Participants were asked to attend to the visual stimuli and press a button with their right index finger at the end of each visual stimulus, and to ignore the auditory stimuli.

### MRI acquisition parameters

All fMRI scans were acquired using a 3.0 Tesla Achieva Philips scanner, with a ${\text{T}}_{2}^{*}$-weighted echo-planar imaging (EPI) sequence (TR/TE = 1,000/20 ms; flip angle = 72°; FOV = 240 × 240 mm; matrix size = 80 × 80; voxel size = 3.0 × 3.0 × 5.5 mm; 18 axial slices). The duration of the tb-fMRI scans was 6 min (360 volumes). An accompanying T_1_-weighted magnetization-prepared rapid gradient-echo (MPRAGE) structural image (TR/TE = 6.5/3 ms; flip angle = 8°; FOV = 25.6 × 25.6 cm; matrix size = 256 × 256; voxel size = 1.0 × 1.0 × 1.0 mm; 165 axial slices) was collected for the localization and spatial normalization of the functional data in each participant.

### Analysis of fMRI data

All fMRI data were analyzed using the FSL (V5.0.7) software package. Realignment of the fMRI scans was performed by rigid-body registration of all the volumes to the middle one. Next, slice-timing correction was performed by shifting the time-series for each slice to the instance when the middle slice was acquired. High-pass filtering was performed with a non-linear Gaussian kernel with cut-off frequency of 0.01 Hz. Then three dimensional spatial smoothing was performed with the full width half maximum (FWHM) of 5 mm (Smith and Brady, 1997). Spatial normalization was performed by rigid-body registration of the first fMRI volume to its T_1_-weighted structural image and then by non-linear registration of the structural image to the MNI template. Finally, intensity normalization was performed by global scaling of the data to have a median of 10^4^. First-level GLM analysis was performed by modeling the fMRI data with three predictors of interest, which were obtained by convolving the canonical HRF with the timing (zero-one boxcar function) of the visual, and audio stimulation as well as motor response (Boynton et al., 2012). The results fed into a second-level analysis to derive group maps of the activations and deactivations using a mixed-effects modeling technique implemented in FSL (Woolrich et al., 2004). The residual of the first-level GLM analysis was then added to the fMRI temporal mean volume to generate the residualized fMRI dataset.

### Independent component analysis

We used ICA to extract the spatial extent and temporal time-course of the FC networks. The same preprocessed data that were fed into the first-level GLM analysis were also fed into a multivariate exploratory linear optimized decomposition into independent components (MELODIC) analysis with temporal concatenation of all subjects (Beckmann and Smith, 2004). The residualized fMRI data were also fed into the MELODIC analysis to perform group level ICA with temporal concatenation. Subject-wise ICA was performed using MELODIC on pre-processed but not spatially normalized fMRI data as well as residualized fMRI data in the subjects' native space. The number of the ICs was estimated automatically using the Laplace approximation to the Bayesian evidence of the model order (Minka, 2000). In order to generate one single IC that corresponded to both significantly activated and deactivated regions, we manually lowered the number of IC to as low as 20 components to examine whether or not the visual network and DMN would get combined to generate a single FC network.

### Obtaining the equivalency interval

Any additional processing of the fMRI data tends to slightly alter the results of the ICA. However, this alteration should not exceed the *equivalency interval* in which the natural variability in the temporal and spatial characteristics of the extracted networks can occur. For instance, removing the task-related variability should slightly alter the temporal and spatial characteristics of the FC networks even if the network fluctuations are completely independent from the task time-course. However, the alteration cannot exceed the *equivalency interval* which needs to be obtained by generating the null distribution for such alterations. To generate the null distribution for removing un-related variability we first permuted the time-course of the tasks across all participants and re-ran the first-level analysis. Each subject had a unique task timing for visual and audio stimulation, thus the results of first-level GLM analysis with permuted task timing should detect no significant activation/deactivation for any of the participants. Next, the residual of the GLM analysis with the permuted task timing was added to the fMRI mean volume to generate the residualized fMRI dataset. The difference here is that the residualizing process now removes only random and task-irrelevant variance from the fMRI data. Performing group ICA and subject-wise ICA on the residualized fMRI data with respect to permuted task timing should generate very similar networks as those obtained from the original fMRI data. Comparison between the temporal and spatial characteristics of the obtained network before and after residualizing of permuted task timing will give us the distribution of the natural alteration in the extracted networks due to any random residualizing. Any significant deviation from this natural variability should be interpreted as true and significant change in the temporal time-course or spatial pattern of the extracted networks. We used simple *Pearson* correlation coefficients (*Pcc*) to determine the similarity between the two time-courses or spatial patterns of each network.

## Results

**Figure 1:**
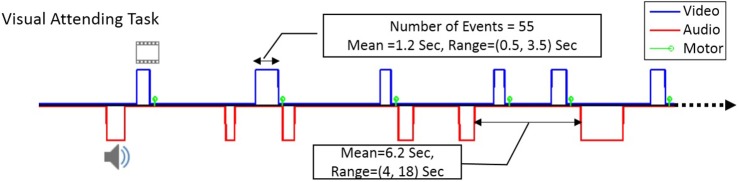
The time course of the visual-motor task in our event-related fMRI task paradigm. The blue line shows typical timing for visual stimuli and the red line shows typical timing for audio stimuli. The green bars represent the time of the motoric response to the stimulus. There were at least 55 visual as well as 55 audio stimuli in each run, with mean duration of 1.2 s (ranging from 0.5–3.5 s).

Figure 1 shows the design and timing of the fMRI paradigm that was used in this study to show the consistency of DMN FC during task performance. As shown, we used an event-related design with at least 55 visual (blue) and 55 audio (red) events of stimulation that were presented to the participants with random onsets and durations. The green bars illustrate the instance when each response was made.

### Group-level analysis with general linear modeling

**Figure 2:**
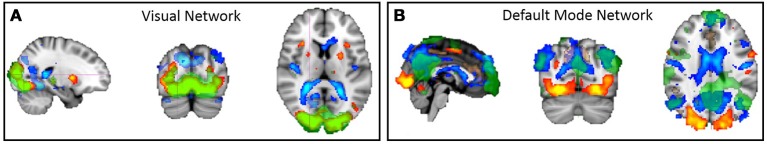
The network of activated/deactivated regions during a simple visual-motor task obtained by GLM group-level analysis. Positive BOLD response is thresholded at *z* > 3 and color-coded with hot color (red to yellow corresponding to *z* = 3 to *z* = 8) and negative BOLD response is thresholded at *z* < −3 and color-coded with cold color (blue to light-blue corresponding to *z* = −3 to *z* = −8). The green overlay is the spatial pattern of two separate functional connectivity networks extracted by ICA, and thresholded at *z* > 3: **(A)** one overlapping with positive BOLD response in the primary visual cortex, and **(B)** another overlapping with negative BOLD response in the DMN regions. Three orthogonal and most informative slices are selected for this illustration.

Using GLM analysis we obtained the pattern of task-evoked positive BOLD response (activation) and negative BOLD response (deactivation) in response to visual stimulation. Figure 2 illustrates the significance of group-level activations with hot colors (red to yellow) and deactivations with cold colors (dark-blue to light-blue) using z statistics. Activation and deactivation maps are overlaid on three orthogonal and most informative slices of the brain in Figures 2A and 2B, respectively. As expected, visual stimulation generates a bilateral positive BOLD response (activation) in the primary visual cortex and negative BOLD response (deactivation) in DMN regions. The auditory stimulation and motor response also resulted in significant bilateral activation in the primary auditory cortex and significant unilateral activation in the contralateral motor cortex, respectively (results are not shown). As seen in Figure 2, there were also significant negative BOLD response in bilateral ventricular regions.

### Group-level analysis with ICA

Using group-level ICA analysis with temporal concatenation of the fMRI data in the same dataset we obtained FC networks during task performance. Group-level ICA detected 105 independent components (IC); the 2nd component accounted for 1.13% of explained variance and showed a high degree of spatial overlap with activated regions in the primary visual cortex (shown as green overlap in Figure 2A), and the 17th component accounted for 1.02% of explained variance and showed a high degree of spatial overlap with the DMN deactivated regions (shown as green overlay in Figure 2B). The time-course of the visual network predicted the time-course of the task (*r* = 0.537, *df* = 28, *p* = 0.001) whereas the time-course of the DMN was not related to task timing (*r* = 0.075, *df* = 28, *p* = 0.34). We could not find any IC that showed a high degree of spatial overlap with both activated and deactivated regions, even when we force ICA analysis to produce many fewer components (as few as 20 components).

### Group-level analysis with ICA on residualized fMRI data

To further examine the relationship between FC networks and task-evoked BOLD response in DMN regions we removed task-related variability from the fMRI data and re-ran the group-level ICA. This time the group-level ICA detected 100 independent components (IC); the 3rd component accounted for 1.17% of explained variance and showed a high degree of spatial overlap with the positive BOLD response in primary visual cortex, and the 22th component accounted for 1.06% of explained variance and showed a high degree of spatial overlap with the negative BOLD response in the DMN regions.

Removing task-related variability altered the temporal characteristic of the visual network such that its similarity to the original time-course dropped to 0.792, measured by *Pcc*, whereas for the DMN it remained relatively high at *Pcc* = 0.9. On the other hand, removing task-related variability caused a subtle change to the spatial pattern of the visual network such that its similarity to the original pattern was as high as 0.97 using *Pcc* or 0.83 using the Dice overlap measure for voxels with significance level of *z* = 3 or higher. Strikingly, this spatial similarity for DMN dropped to 0.69 using *Pcc* or 0.51 using Dice. The high temporal similarity of the DMN and low temporal similarity of visual networks were expected due to the fact that only the temporal time-course of the visual network was related to the task time-course. However, the finding of high spatial similarity of the visual network before and after residualizing, and low spatial similarity of the DMN before and after residualizing was somewhat unexpected. Since the time-course of the DMN activity was not related to task timing, one would have expected that removing task-related variance should have no effect on the temporal and spatial characteristics of the DMN. On the other hand, since almost 30% of the variance in the time-course of the visual network was accounted for by the time-course of the task, one might have expected that removing task-related variability would significantly alter the spatial characteristics of the visual network as well. One methodological challenge in group-level ICA is the spatial normalization step required for temporal concatenation of all participants' fMRI data. We have previously reported the deterioration of the FC networks (Razlighi et al., 2014) as well as task-evoked BOLD response (Liu et al., 2017) due to inaccuracy in this spatial normalization step. To overcome this methodological challenge we also performed a subject-level ICA analysis. Subject-wise ICA method circumvents the need for inaccurate spatial normalization and can be performed in participant's native space, however it requires tedious manual identification of different ICs for each participant.

### Subject-wise ICA

**Figure 3:**
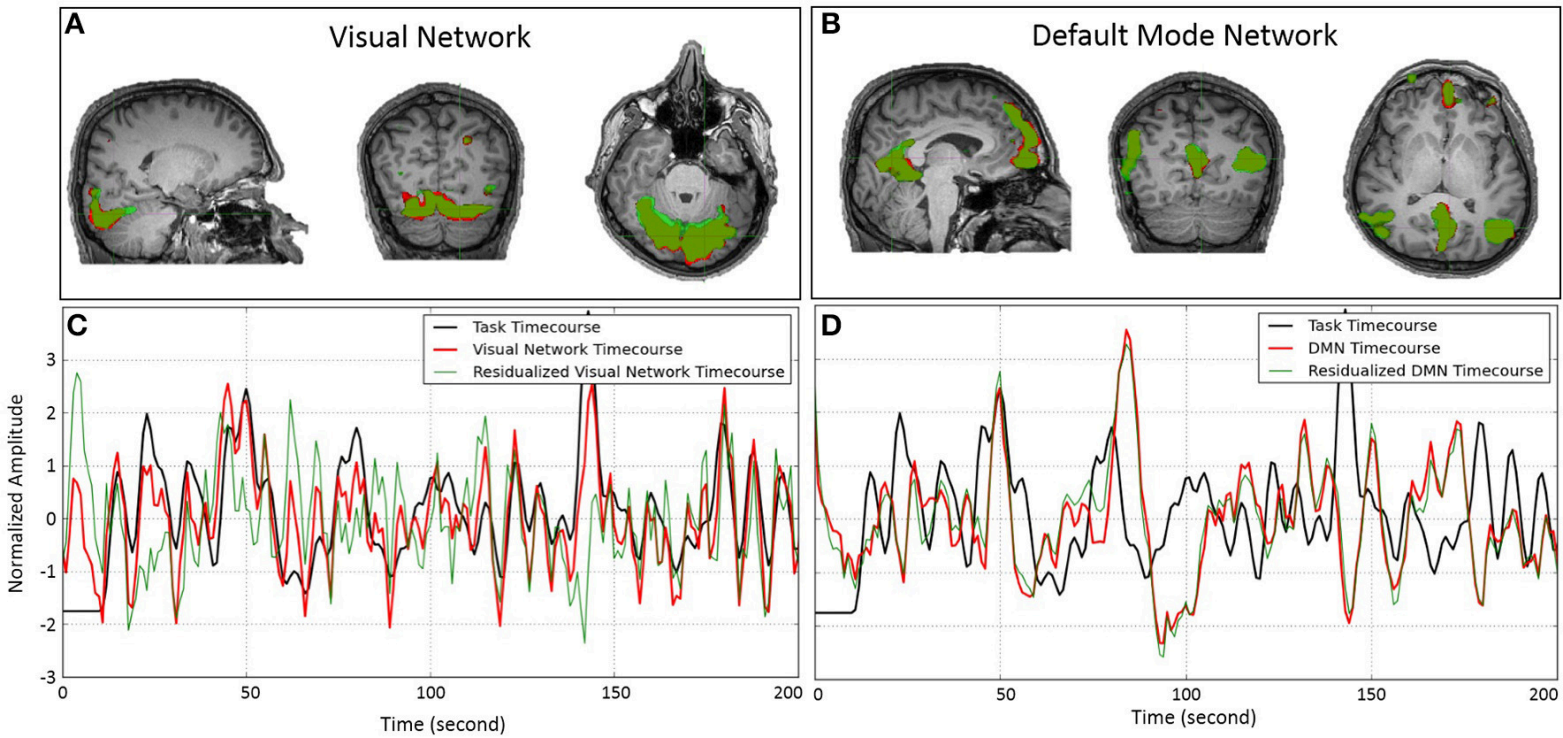
Spatial pattern and temporal time-course of the visual network **(A,C)** and DMN **(B,D)** obtained by ICA on a single subject before (red) and after (green) removing task-related variability from the fMRI data. The spatial overlaps in **(A,B)** are delineated in darker green color. **(A,B)** Show almost a perfect match between the spatial patterns of the networks before and after removing the task-related variability. **(C,D)** Illustrate the temporal similarity of the networks' time-course before (red) and after (green) removing task-related variability. The time course of the task is depicted in black. While removing task-related variability significantly alters the time-course of the visual network, it has almost no effect on the time-course of the DMN.

We performed subject-wise ICA on both original fMRI data as well as residualized fMRI data with respect to task-related variability. Performing subject-wise ICA on original fMRI data resulted in an average of 79 ICs per participant (range: 62–99); the same analysis on residualized fMRI data resulted in an average of 71 ICs per participant (range: 57–90). We were able to identify visual networks in every participant in which the time-course always significantly predicted the task time-course (on average z-statistics = 12.24 ranging from 6.2 to 16.37). In all participants but one, we were able to identify the DMN, in which the time-course of activity was not related to the time-course of the task (except in a couple of cases in which the time course predicted the task timing, but in opposite directions—in one participant with *z* = 3.39 and the other with *z* = −3.65). Figure 3 illustrates the spatial and temporal similarity of the visual network (Figures 3A,C) and DMN (Figures 3B,D) in a typical participant before and after removing task-related variability. The spatial extent of the FC network is depicted in red for original fMRI data, in green for residualized fMRI data, and in dark-green for regions of overlap. Figure 3 also plots the time-course of the visual task (in black), and the FC networks before (in red) and after (in green) removing task-related variability for the visual network (Figure 3C), and the DMN (Figure 3D). As evident in the graphs, the time-course of the visual network significantly predicts the task (*z* = 16.08, *p* < 10^−8^), whereas the time course of the DMN is not related to the task (*z* = −2.24, *p* = 0.98). Removing task-related variability completely changes the time-course of the visual network (*Pcc* = 0.66), while the spatial similarity remains relatively high (*Pcc* = 0.82). On the other hand, removing task-related variability had negligible effect on the time-course (*Pcc* = 0.98) as well as spatial pattern (*Pcc* = 0.93) of the FC in the DMN. While the results in this single participant suggest that task-evoked negative BOLD response does not alter FC of the DMN, it is crucially important to generate the null distribution to assess the significance of our findings.

### Generating the null distribution

**Figure 4:**
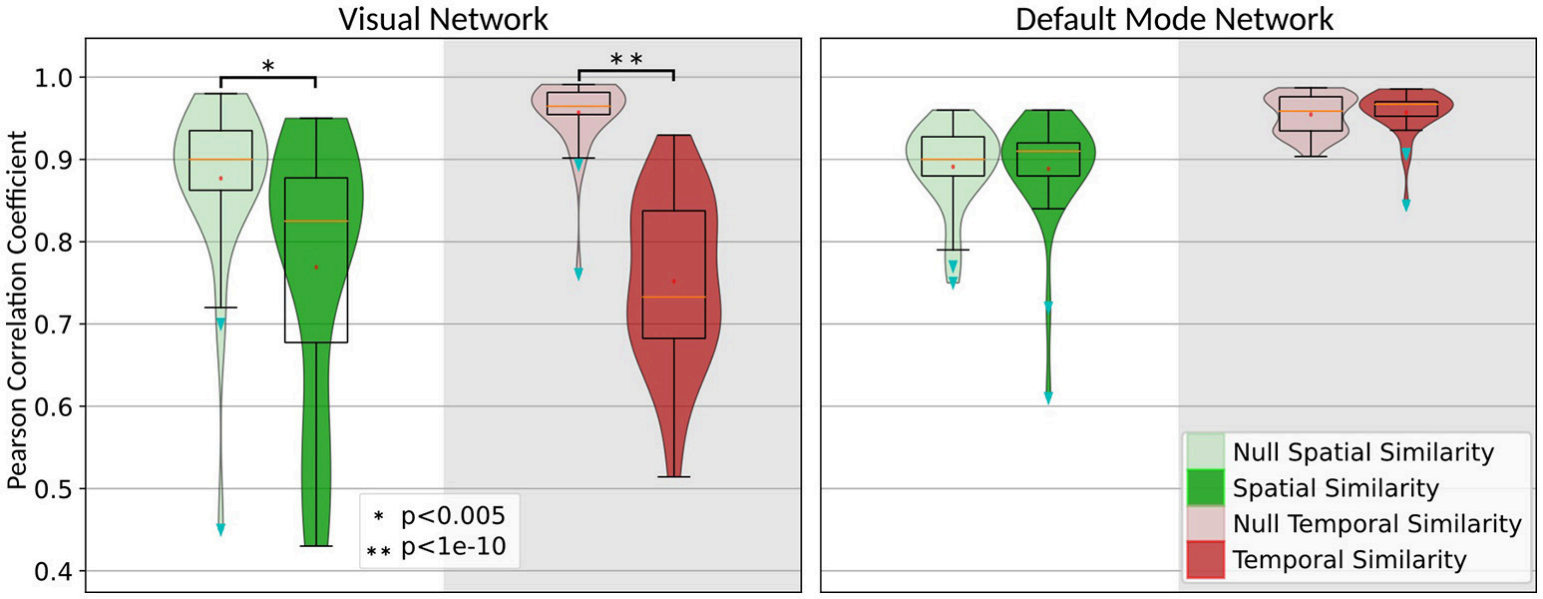
Illustration of the significance of the alteration in the spatial (green) and temporal (red) characteristics of the visual network and DMN along with their null distributions (light green and light red).

The statistical assessment of the significance of our findings requires generating a null distribution to obtain the *equivalency interval* for spatial and temporal alteration of the IC before and after removing the task-related variability. We had generated this distribution by permuting the task timing between subjects and performing the first-level analyses. In this case, the temporal and spatial similarity of the extracted IC before and after residualizing should give the range in which the *Pcc* can fluctuate as the natural variation due to removing un-related/random task variability. Figure 4 shows the distribution of the spatial and temporal similarity for both the visual network as well as the DMN. Next to each null distribution is the distribution of the similarity that we had obtained for spatial and temporal similarity of the visual network and DMN. Performing a simple pair-wise student t-test will determine whether the observed alteration in the spatial and temporal characteristics of the network is significant, or if they are within the *equivalency interval* obtained from the null distribution. As it seen in Figure 4, removing task-related variability significantly altered temporal time-course (*t* = 10.17, *p* < 10^−13^) and spatial pattern (*t* = 3.057, *p* < 0.003) of the visual network whereas both temporal time-course (*t* = −0.35, *p* = 0.73) and spatial pattern (*t* = 0.15, *p* = 0.88) of the DMN remained intact despite detecting a robust and overlapping negative BOLD response within the same regions.

## Discussion

Using univariate GLM analysis, we first detected the brain regions with positive BOLD response (activation) and negative BOLD response (deactivation) for a simple visual-motor task. Then we extracted FC networks by applying a multivariate technique (e.g., ICA) on the same set of fMRI data. While the regions with positive and negative BOLD response were obtained simultaneously in response to the same stimuli, we could not find a single IC that spatially corresponded to both areas of activation and deactivation. Instead, two separate IC were detected: one corresponding to the activated regions, and another corresponding to the deactivated regions. This separation remained even when we reduced the number of ICs to 20, suggesting that the time-courses of the BOLD response are different for activated and deactivated regions, even though they are both correlated with the time-course of the task. Furthermore, the time-course of the IC that spatially corresponded to activated regions (primary visual cortex) were highly correlated with the time-course of the task, whereas the time-course of the IC that spatially corresponded to deactivated regions (DMN) were not correlated with the time-course of the task. In other words, while the fMRI signal in DMN regions was correlated negatively with the task timing, the IC extracted from the same regions using FC analysis was not temporally correlated with the task. This suggested a disassociation between the task-evoked negative BOLD response and an FC network in the DMN regions. To quantitatively examine this disassociation, we then performed subject-wise FC analyses by applying an ICA to the original tb-fMRI data as well as residualized tb-fMRI data with respect to all task-related variability. We demonstrated that removing task-related variability from fMRI data significantly altered spatial and temporal characteristics of the visual network, which corresponded to the activated regions, whereas both spatial and temporal characteristics of the DMN, which corresponded to deactivated regions, remained intact. This finding demonstrates that task-evoked negative BOLD response in the DMN regions does not alter its intrinsic FC. To the best of our knowledge, this disassociation between the negative BOLD response and FC in the DMN has not been shown previously. This finding becomes even more interesting when compared to its counterpart in the activated regions (primary visual cortex), where task performance significantly altered both spatial and temporal characteristics of the FC network.

Current studies investigating the relationship between task-evoked BOLD response and rs-FC networks indicate a significant overlap between the two networks (Toro et al., 2008; Smith et al., 2009; Power et al., 2011; Crossley et al., 2013; Di et al., 2013). However, the effect of task performance on the temporal characteristics of the FC networks is not completely understood. Some studies claim a close correspondence between rs-FC and tb-FC networks (Greicius et al., 2003; Fair et al., 2007; Fox and Raichle, 2007) to the degree that they even dismiss the need for tb-fMRI studies to map the functional organization of the brain (Fair et al., 2007). Other studies, however, report subtle alteration in the coherence of within as well as across FC networks due to task performance (Cole et al., 2014; Gerchen et al., 2014; Krienen et al., 2014; Spadone et al., 2015; Gratton et al., 2016; Kaufmann et al., 2017). Our findings suggest that depending on the positivity or negativity of the BOLD response, different mechanisms may underlie the relationship between the task-evoked BOLD response and the overlapping FC network. This is an important finding because one of the most studied FC networks, the DMN, is also the most commonly-reported site of the negative BOLD response.

Another important yet unanswered question in the field is whether task-evoked BOLD response and FC network are both manifestations of the same neurophysiological process or each represent a distinct process. Some studies have hypothesized that brain activity is comprised of two separate components: spontaneous and task-evoked activities (Arieli et al., 1996; Fox et al., 2006). This has been shown by electro-encephalography (EEG) (Scherrer, 1976; Mayhew et al., 2013), electrophysiological recording (Arieli et al., 1996) and recently with fMRI (Fox et al., 2006). Translating such a hypothesis to fMRI means that FC networks are representative of the spontaneous activity, and task-evoked BOLD response is thought to represent task-related activity (Fox et al., 2006). In the present study, the disassociation between task-evoked BOLD response and FC clearly support this hypothesis for the DMN; however, our findings in the visual network contradict this hypothesis. Here we show that visual network FC fluctuation, as a measurement of its spontaneous activity, is correlated with the time-course of the task, suggesting it cannot purely be a measurement of spontaneous brain activity. One possibility that might explain this disagreement in the visual network is that the spatial ICA method that we used to extract FC networks, which is the most commonly used method in the field, is not suitable for extracting spatially overlapping networks, as has been reported previously (Calhoun et al., 2001; Smith et al., 2012). By definition, the spatial ICA method searches for spatially independent components in the fMRI data. Further examination with temporal or spatio-temporal ICA techniques is required to test such a possibility. This issue with spatial ICA seems to be less disparaging with the negative BOLD response in the DMN regions, since the hemodynamic response for negative BOLD seems to be different for each node of the DMN (Lustig et al., 2003). While GLM analysis is less sensitive to such variations in the BOLD response, spatial ICA tends to detect those variations and effectively separate them from the FC fluctuation. Therefore, the extracted time-course for the DMN is not correlated with the task time-course. Comprehensive simulation is needed to test this possibility on synthesized fMRI data, in order to determine whether temporal or spatio-temporal ICA methodology is more appropriate for separating spatially overlapping networks in real fMRI data.

One methodological challenge in extracting the FC networks is the parcellation scheme used in the pre-processing of the fMRI data. Many different parcellations of the human brain have been utilized in studies investigating FC networks (Fischl et al., 2004; Power et al., 2011; Yeo et al., 2011; Craddock et al., 2012; Shirer et al., 2012; Wig et al., 2014; Glasser et al., 2016). These parcellations are not only fundamentally different in their underlying segmentation/registration techniques, they also differ in their parcel sizes, numbers, and shapes. These differences make the comparison of the results of different studies a challenging process. To overcome this issue we used ICA to automatically extract the FC networks without any presumption on their size and shape. The number of ICs was also extracted automatically (Minka, 2000). The only problematic pre-processing step associated with group ICA technique is its requirement for spatial normalization. In fact, our initial attempt to use group ICA produced inconclusive results for DMN. While removing task-related variability had a subtle alteration in the temporal time-course of the network (*Pcc* = 0.90), the spatial pattern of the network changed drastically (*Pcc* = 0.69, or Dice = 0.52 for voxels with *z* ≥ 3), suggesting a significant task-based alteration in the spatial pattern of the DMN (see the null distribution in Figure 4). Considering the relatively stable temporal time-course of the DMN, we suspected that the drop in the spatial correlation could be a result of inaccuracy in the spatial normalization process. We have shown previously that spatial normalization accuracy using a state of the art non-linear registration method is at 53% for cortical regions (Razlighi, 2016). Therefore, we switched to subject-wise ICA and performed the rest of the study in subject native space. While the subject-wise ICA circumvented the need for spatial normalization, it required the tedious process of manual identification of different FC networks, which for some subjects it could be challenging. Fortunately, we were able to identify all visual networks and, except for one participant, we were able to identify the DMN in all subjects as well. As we have demonstrated, repeating the analysis in the subjects' native space showed that task-evoked BOLD response had no effect on the FC of the DMN. This finding suggests that the error introduced by substandard spatial normalization could potentially be larger than the effect of task performance on the FC networks. Thus extra caution should be warranted when investigating small alterations in the FC networks.

There is a consensus in the field that functional connectivity fluctuations are concentrated in the lower frequency range (<0.1 Hz); however, the frequency spectrum of the task-evoked negative BOLD response has not been fully investigated. Most of the existing research on the negative BOLD response assumes the same positive BOLD response for negative BOLD as well, yet there is evidence that each node in the DMN has a unique BOLD response with higher frequency components (Lustig et al., 2003; Hayden et al., 2009). Therefore, we tried to increase the sampling rate (TR) of our fMRI data as much as possible to capture the higher frequency components in the negative BOLD response. We first reduced the TE to 20 ms, which is beneficial in alleviating the signal drop-off in the medial pre-frontal regions (one of the main nodes in the DMN), and then increased the slice thickness to 5.5 mm which is beneficial for improving signal to noise ratio (SNR) in fMRI time-series. By increasing the SNR, we also expected to improve our detection power for the negative BOLD response, since it is a weaker signal in comparison to the positive BOLD response. Nonetheless, we used a spatial smoothing kernel with FWHM = 5 mm in the pre-processing pipeline which theoretically has the same effect on the SNR for smaller voxel sizes. Having said that, we emphasize that a replication of our results, particularly the spatial similarities of the functional connectivity networks, seems to be warranted in the future with faster sampling rate and higher spatial resolution, which is now available with multi-band image acquisition techniques (Breuer et al., 2005; Setsompop et al., 2012).

Most recent studies investigating the effect of task performance on FC networks throughout the whole brain report only subtle alterations in their FC strength (Cole et al., 2014; Krienen et al., 2014; Gratton et al., 2016). These studies usually assess the stability of the FC networks by comparing the correlation matrices obtained for rs-fMRI data and the task-variability-removed tb-fMRI data. In other words, they investigate any task-related alteration in the second order statistical moment, while we assessed the task-related alteration in the time-course of the shared variance among actual MR signals which is given by ICA. While these two methods might seem to target different characteristics of the FC networks, we should emphasize that the correlation coefficient is directly related to the standard deviation of the time-course of the shared variance. Thus, alteration in the correlation coefficient is not possible without changing the time-course of the shared variance. Furthermore, the significance of the subtle change in the correlation matrix has been determined by parametric statistics in the existing studies. However, what is not reported in these studies is the subtle changes between the correlation matrices obtained from two repeated rs-fMRI scans. A permutation between repeated rs-fMRI scans can be used to generate the null distribution to assess the significance of the observed subtle changes in these studies. Therefore, in the current study, we have generated the null distribution by permuting the time-course of the tasks across different subjects, making sure there is no correlation between the time-course of the task for different subjects. Essentially, we examined the effect of removing an unrelated time-course from the fMRI data and quantified the effect of such alteration in the extracted functional networks. A simple pair-wise student *t*-test determined that the observed alteration in the visual network was a significant change, whereas the detected subtle changes in the DMN were just an effect of natural variability and artifacts in the fMRI data processing pipeline.

## Conclusion

The evidence presented in this work clearly establishes a disassociation between the DMN functional connectivity and its task-evoked negative BOLD response. This finding becomes more interesting when compared with its counterpart in the task-evoked positive BOLD response. Task performance modulated the time-course of the FC network when it is extracted from regions with positive BOLD response; suggesting that depending on the BOLD response being positive or negative, the mechanism underling the relationship between BOLD response and functional connectivity may differ significantly. We conclude that the task-evoked negative BOLD response in the DMN regions is a separate and distinct measurement from the DMN regions, which is fundamentally different from its functional connectivity. Therefore, it could be considered as a separate imaging biomarker that can be utilized in studies investigating the relationship between the brain and cognition.

## Author contributions

The author confirms being the sole contributor of this work and approved it for publication.

### Conflict of interest statement

The author declares that the research was conducted in the absence of any commercial or financial relationships that could be construed as a potential conflict of interest.
